# NICD-mediated notch transduction regulates the different fate of chicken primordial germ cells and spermatogonial stem cells

**DOI:** 10.1186/s13578-018-0238-y

**Published:** 2018-06-19

**Authors:** Qisheng Zuo, Chen Zhang, Kai Jin, Jin Jing, Changhua Sun, Mahmoud F. Ahmed, Jiuzhou Song, Yani Zhang, Guohong Chen, Bichun Li

**Affiliations:** 1 Key Laboratory of Animal Breeding Reproduction and Molecular Design for Jiangsu Province, College of Animal Science and Technology, Yangzhou University, 88 South University Ave, Yangzhou, Jiangsu 225009 People’s Republic of China; 2 Department of Animal & Avian Sciences, University of Maryland, Baltimore, MD 20741 USA; 3 College of Veterinary Medicine, Suez Canal University, Ismailia, 41522 Egypt; 4 Joint International Research Laboratory of Agriculture and Agri-Product Safety of Ministry of Education of China, Yangzhou University, 88 South University Ave, Yangzhou, 225009 Jiangsu People’s Republic of China

**Keywords:** Primordial germ cells, Spermatogonial stem cells, Notch signaling, RNA-seq, Transduction

## Abstract

**Background:**

Notch signaling is mainly regulated by Notch1 during development of chicken germ stem cells; however, the molecular mechanisms that contribute to generation of these germ stem cells have not been thoroughly investigated.

**Results:**

In our studies, Overexpression of the Notch1 NICD promoted development of the reproductive ridge, but inhibited the formation of seminiferous tubules. The formation efficiency of PGCs in the reproductive ridge following overexpression of NICD (7.5% ± 0.11) was significantly higher than that (4.9% ± 0.17, *p *< 0.05) following inhibition of NICD, While the formation efficiency of spermatogonial stem cells (SSCs) in the testes (12.7% ± 0.08) was significantly lower after NICD overexpression than that after inhibition of NICD (16.3% ± 0.16, *p *< 0.05). Using co-immunoprecipitation, we found that this anomaly stemmed from the reversal of dissociation of the Notch-regulated transcription factor CBF-1/RBP co-suppression complex during the differentiation of PGCs into SSCs. This dissociation of the CBF-1/RBP co-suppressing complex during the differentiation of ESCs into PGCs resulted in the release of HDAC1 and HDAC2 and the recruitment of mastermind-like 1 to form a coactive complex to promote the expression of the downstream transcription suppressor hairy/enhancer of split-1. Dynamic expression of transducin-like enhancer of split 3, TLE4, and C-terminal binding protein 2 during further differentiation of PGCs inhibited the dissociation of the CBF-1/RBP co-suppression complex and inhibited the expression of the downstream genes.

**Conclusions:**

In summary, Notch signaling plays diametrically opposing roles during normal development of chicken PGCs and SSCs, and these functions was determined by the expression of NICD, changes in the CBF-1/RBP complex composition, and histone modification.

**Electronic supplementary material:**

The online version of this article (10.1186/s13578-018-0238-y) contains supplementary material, which is available to authorized users.

## Background

Although research on in vitro differentiation of germ cells from embryonic stem cells (ESCs) has advanced considerably, several gaps remain. Specifically, low repeatability and efficiency in induction of germ cell differentiation and a lack of clarity about the molecular regulation of this process challenge progress in this field [1]. Further studies of the mechanisms of reproductive cell regulation are warranted, and new methods to induce ESC differentiation into germ cells are needed. Recent findings describe the signaling pathways such as Notch, TGF-β that participate in differentiation regulation of ESCs into germ cells [2–4]; however, the mechanism of action of the components of these pathways is not known.

Several studies have investigated aspects of the Notch signaling pathway. Dallas et al. found that this pathway plays a negative role in male *Drosophila* ESCs ecology [5]. Braydich-Stolle maintained spermatogonial stem cells (SSCs) with brain-derived neurotrophic factor and detected increased expression of NUMB, a protein that causes the degradation of the intracellular domain of Notch1 (NICD), as well as the inhibition of Notch target genes. Thus, Notch1 may function to promote the differentiation of SSCs [6]. Garcia et al. reported that deletion of the Notch receptor Jagged 1 leads to the development of the mouse ovarian follicles during different stages of the estrous cycle [7]. By screening mutations in Notch, Huang et al. found that a decrease in Notch1 expression upregulated hairy/enhancer of split (HES-5) and downregulated Neurog3, a SSCs differentiation marker [8].

Despite many in-depth studies on Notch signaling and germ cell differentiation [9–13], the roles of these signaling molecules in vivo germ cell differentiation have not been elucidated. In particular, the role of Notch signaling in the in vitro differentiation of male germ cells from ESCs is unclear. Here, we found that the NOTCH signaling pathway is involved in the regulation of male germ cell differentiation. We then studied the specific function of Notch signaling in the generation primordial germ cells (PGCs) and SSCs and investigated the specific molecular mechanisms. These results were validated using an in vitro induction model and high-throughput sequencing. We expect that such a study may provide an experimental basis for the construction of a regulatory network for chicken male germ cell differentiation and for elucidating the mechanism of germ cell formation.

## Results

### Notch1 regulates gonadal development in chicken germ cells

**Fig. 1:**
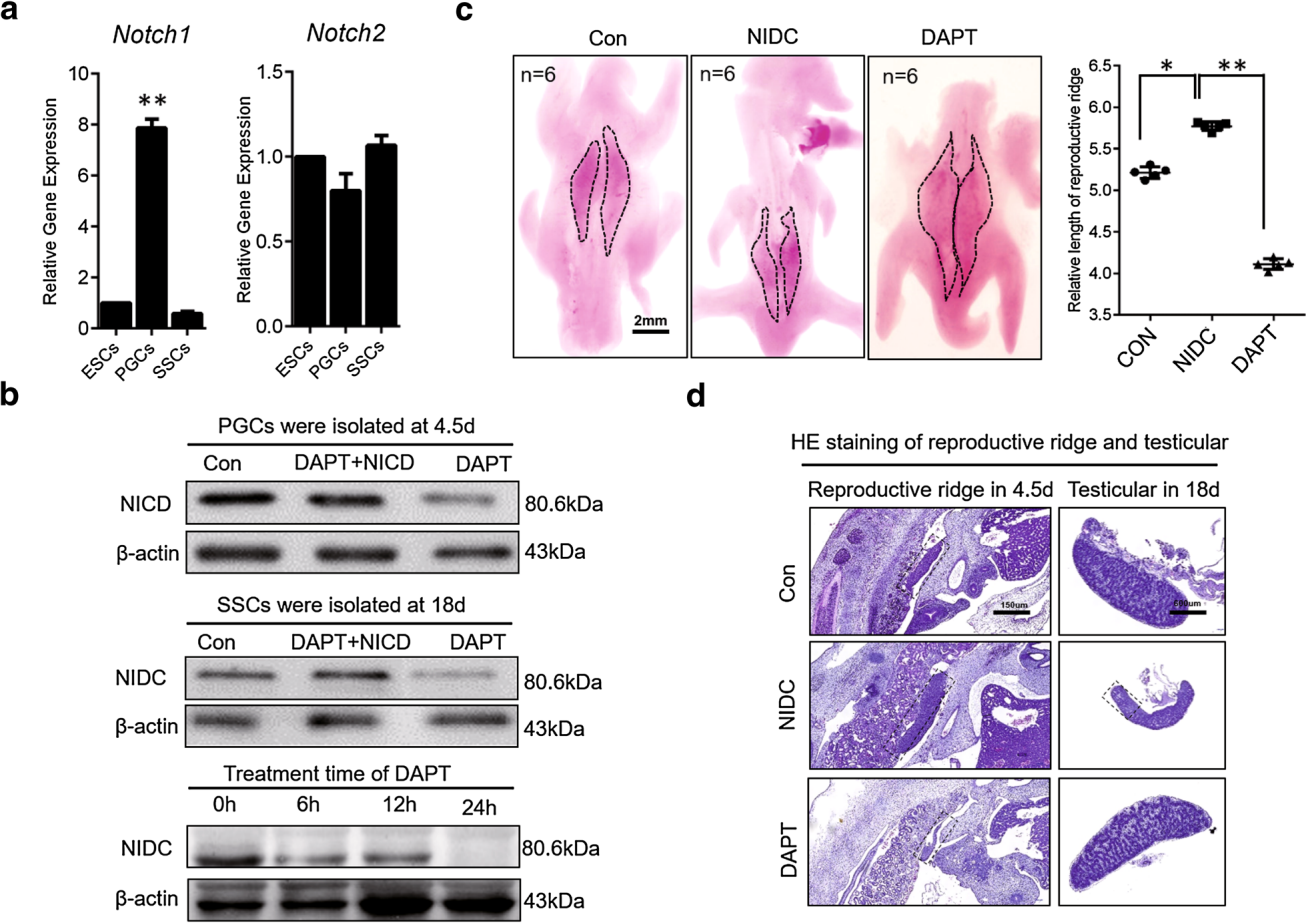
Notch1 is a unique Notch receptor in chicken germ cells that regulates gonadal development. **a** The expression of Notch1 and Notch2 in ESCs, PGCs, and SSCs was detected by qRT-PCR. The expression of Notch1 in the three types of cells was significantly different and highest in PGCs. No significant difference in the expression of Notch2 in the three cell types was observed. **b** Notch1 protein expression was detected by western blot after overexpression and inhibition of NICD. DAPT was used to inhibit the expression of NICD protein level, whereas overexpression of NICD was able to rescue Notch1 protein expression. **c** Morphological changes in reproductive ridge development were observed after NICD overexpression or inhibition. Inhibition of NICD hindered the normal development of the reproductive ridge. Scale bar: 2 mm. **d** Testicular development changes were detected by H&E staining after Notch1 overexpression or inhibition. Scale bar: 150 µm

The Notch signaling pathway is highly conserved throughout evolution and is involved in the cell differentiation process [14] through the functions of Notch1, Notch2, Notch3, and Notch4 [15]. Only Notch1 and Notch2 receptors were detected during chicken germ cell differentiation. Notch1 expression was significantly altered during germ cell formation, while Notch2 expression was not (Fig. 1a). Notch1 was strongly expressed in PGCs but only weakly expressed in ESCs and SSCs (Fig. 1a left). These observations suggest that the Notch signaling pathway may only be regulated by Notch1 during germ cell formation in chicken.

To further study the specific functions of the NICD receptor and Notch signaling pathway in chicken germ cell formation, we treated the chicken PGCs and SSCs with the Notch inhibitor DAPT (*N*-[*N*-(3,5-difluorophenacetyl)-l-alanyl]-*S*-phenylglycine t-butyl ester) [16, 17] and pcDNA3.0-NICD. DAPT was able to inhibit NICD completely within 24 h but had no significant inhibitory effect on Notch2 (Fig. 1b, Additional file 1: Figure S1C). We injected DAPT and pcDNA3.0-NICD into chicken embryos. We observed that inhibition of NICD expression indeed affects the development of chicken embryos. Specifically, the inhibition of NICD altered the development of the reproductive ridge and resulted in developmental retardation (Fig. 1c, Additional file 1: Figure S1A, B). Inhibition of NICD actually promoted the development of the testes. We found that the seminiferous tubules suffered from hypoplasia in the testicular slices with NICD overexpression (Fig. 1d, Additional file 1: Figure S1D). Taken together, these results indicate that NICD acts as the sole receptor for the Notch signaling pathway and exerts opposite effects on reproductive ridges and testicular development.

### CBF-1/RBP-dependent notch signaling regulates downstream gene expression by altering histone acetylation

**Fig. 2:**
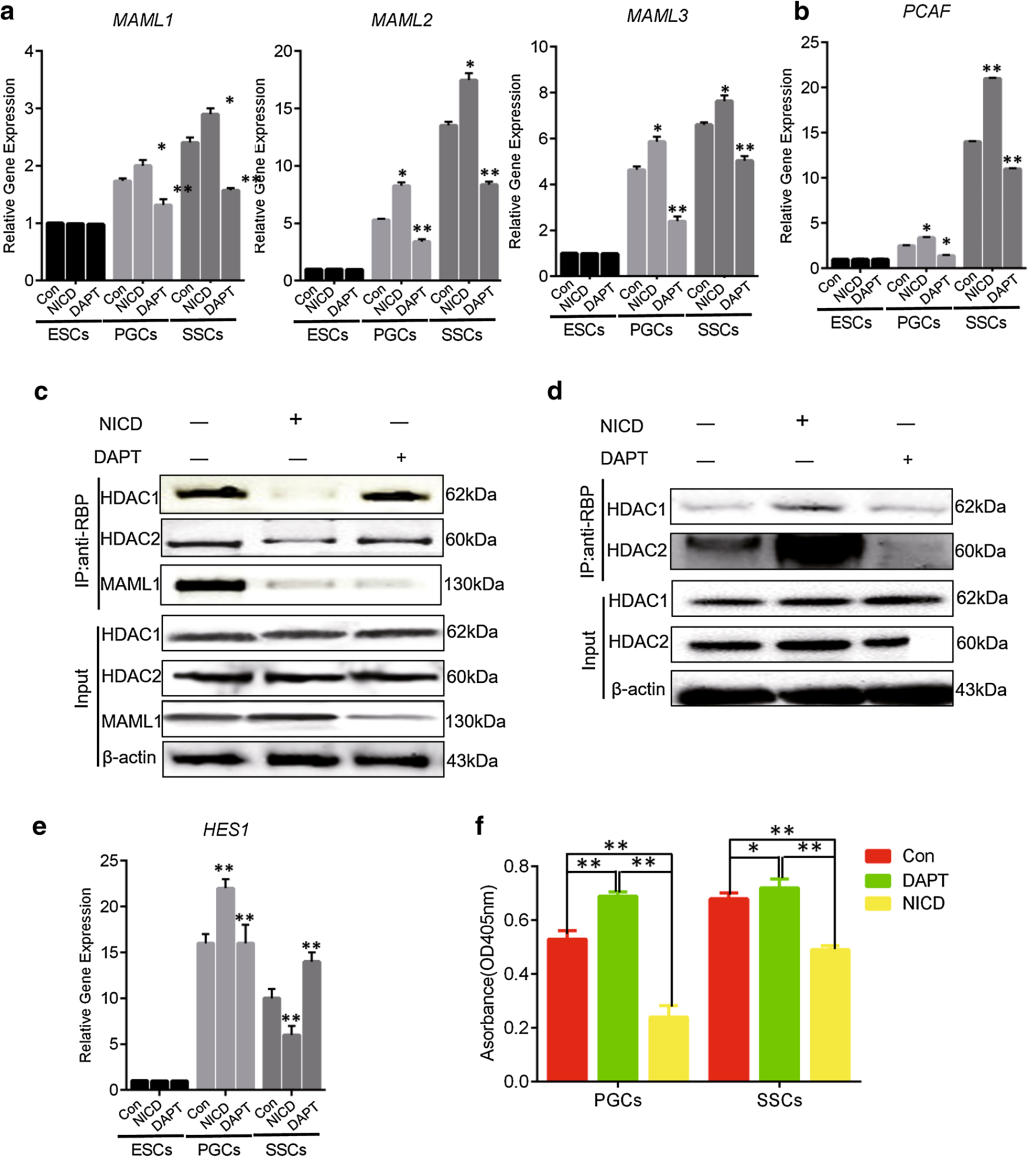
Notch signaling is dependent on CBF-1/RBP and regulates downstream gene expression. **a** The expression of MAML1, MAML2, and MAML3 genes was detected by qRT-PCR. **b** The expression of PCAF was detected by qRT-PCR. **c** Changes in HDAC1, HDAC2, and MAML1 levels in the CBF-1/RBP complex were detected by anti-RBP immunoprecipitation in PGCs with either Notch1 overexpression or inhibition. Β-actin served as the internal reference. **d** Anti-RBP immunoprecipitation was used to detect the changes of HDAC1 and HDAC2 in CBF-1/RBP complex after Notch1 overexpression and inhibition in SSCs. **e** The expression of HES1 was detected by qRT-PCR. **f** Colorimetric results showed that HDACs had significantly lower enzyme activity than SSCs in PGCs; overexpression of NICD could reduce HDACs enzyme activity, while inhibiting NICD could increase HDACs enzyme activity

Classical Notch signaling relies on CBF-1/RBP [18], which is a transcriptional factor that inhibits the expression of downstream genes [19].To explore the transduction of Notch signaling in chicken germ cell differentiation in detail, NICD inhibition and overexpression were performed to inhibit or activate the Notch signal, and downstream signaling molecules were assessed. Expression of mastermind-like (MAML)1, MAML2, and MAML3 as well as expression of the p300/CBP-related factor (PCAF) were significantly upregulated following activation of Notch signaling during the formation of PGCs (Fig. 2a, b). The downstream transcription factor hairy/enhancer of split-1 (HES1) was also upregulated. Expression of the phenomenon, inhibition of Notch1 has the opposite phenomenon (Fig. 2e).

To investigate these effects further, we examined the composition of the CBF-1/RBP complex. We found that activation of Notch signaling resulted in dissociation of co-suppression complexes of CBF-1/RBP, a significant reduction in the enrichment of histone deacetylase (HDAC)1 and HDAC2 in the complex, and formation of co-activated complexes with MAML1 (Fig. 2c). Observation of high expression of PCAF also demonstrated the formation of co-activated complexes (Fig. 2b). The expression of HES1 was promoted by increasing the level of histone acetylation near the transcriptional binding site of HES1, leading to regulation of expression of downstream genes(Fig. 2e).We next transfected DF-1 cells with HES1 and HES5 promoter dual-luciferin reporter vectors and treated the cells with the HDAC inhibitor Trichostatin A (TSA). HES5, a transcription factor downstream of Notch, was not affected by histone acetylation, although both HES1 and HES5 promoters are enriched by RBP as determined by chromatin immunoprecipitation (Additional file 2: Figure S2A, B).Interestingly, with respect to the formation of SSCs, we found that the dissociation process of the CBF-1/RBP co-suppression complex was reversed due to unknown factors. Moreover, the enrichment of HDAC1 and HDAC2 in the co-suppression complex was significantly increased (Fig. 2d), leading to significant reduction in the expression of downstream transcription factors. These results indicate that Notch signaling regulates downstream gene expression by altering histone acetylation levels via dynamic expression of HDAC1 and HDAC2 in CBF-1/RBP complexes.

### Notch signaling positively regulates the formation of PGCs, but negatively regulates the formation of SSCs in vivo

**Fig. 3:**
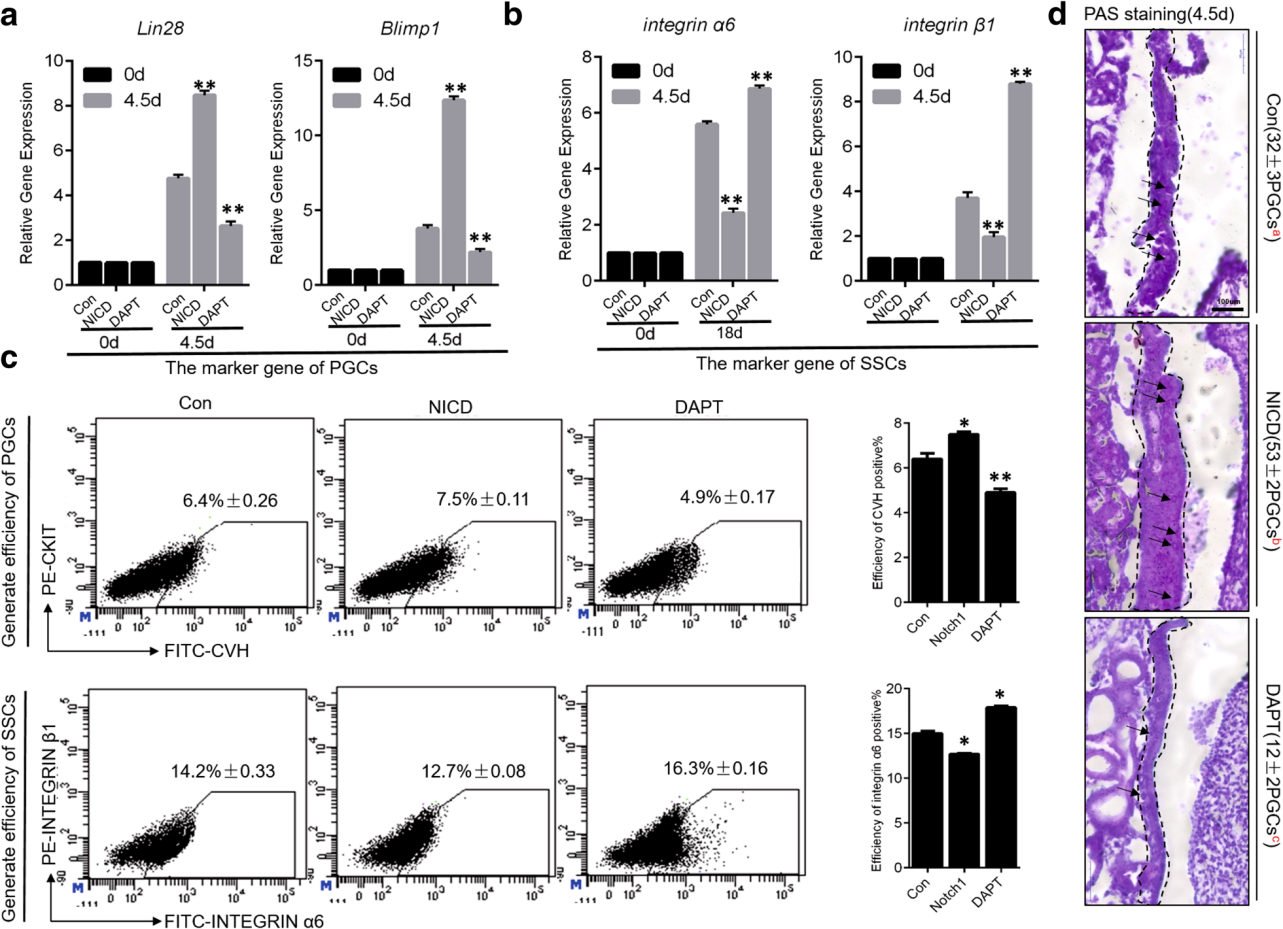
Notch signalingpositively regulates the formation of PGCs and negatively regulates the formation of SSCs in vivo. **a**, **b** The expression levels of PGC and SSC marker genes were detected by qRT-PCR after NICD overexpression and inhibition in the in vitro model. **c** PGCs and SSCs formation efficiency was analyzed by flow cytometry after NICD overexpression and inhibition. **d** PAS staining of PGCs and SSCs following NICD overexpression and inhibition. PAS staining showed that NICD promoted the formation of PGCs, whereas inhibition of NICD expression inhibited the formation of PGCs. Arrow represents PGCs. Scale bar: 100 μm

We next further explored the specific function of Notch signaling during germ cell formation. To this end, we detected the formation of PGCs (4.5 days) [20] and SSCs (18 days) [21, 22] after activation and inhibition of Notch signaling during chick embryo hatching. With respect to PGCs, we found that the activation of Notch signaling significantly upregulated the expression of Lin28 (8.3427 ± 0.23, *p *< 0.05) and Blimp1 (12.4213 ± 0.13, *p *< 0.05), which mark formation of PGCs at 4.5 days. Inhibition of Notch signaling induced the opposite effects on Lin28 and Blimp1 (Fig. 3a). These data indicate that Notch signaling may positively regulate the formation of PGCs. We also analyzed the CVH^+^ CKIT^+^ efficiency (which Used to mark PGCs) during the chicken embryo hatching process following manipulation of Notch signaling. Activation of Notch signaling promoted the formation of PGCs in reproductive ridges and the efficiency of CVH^+^ CKIT^+^ cells (7.5% ± 0.11, *p *< 0.05) was significantly higher than that of embryos that did not have activated Notch signaling(control, 6.4% ± 0.26). On the other hand, inhibition of Notch signaling significantly reduced the number of PGCs in the reproductive ridge, and the efficiency of CVH^+^ CKIT^+^ cells was only 4.9% ± 0.17 (*p *< 0.05 compared to control without Notch inhibition (Fig. 3c top). At the same time, the results of periodic acid Schiff (PAS) [23] staining also demonstrated that Notch signaling enhanced the formation of PGCs, although the morphological development of the reproductive ridge was the same in the presence and absence of Notch activation. The number of PGCs was nearly 30 ± 3 in the control group, but was 53 ± 2 following Notch activation. In accord with our previous observations, the number of PGCs formed after inhibition of Notch signaling was only 12 ± 2 (Fig. 3d). These results indicate that Notch signaling positively regulates the formation of PGCs.

The same phenomenon was not observed during the formation of SSCs. We found that the expression of integrin α6 (2.8461 ± 0.31, *p *< 0.05) and integrin β1 (1.8253 ± 0.44, *p *< 0.05) were significantly downregulated after Notch signaling activation at 18 days. In contrast, suppression of Notch signaling resulted in upregulation of integrin α6 and integrin β1 (Fig. 3a). We also analyzed the efficiency of integrin α6^+^ integrin β1^+^ cell formation following modulation of Notch signaling. Activation of Notch signaling inhibited the formation of SSCs in testicles, and the efficiency of integrin α6^+^ integrin β1^+^ cells (12.7% ± 0.08) was significantly lower than that in embryos without Notch activation (control, 14.2% ± 0.33, *p *< 0.05). Inhibition of Notch signaling, however, significantly promoted the formation of SSCs in the testicles, and the efficiency of integrin α6^+^ integrin β1^+^ cells was 16.3% ± 0.16(Fig. 3b, bottom). Therefore, these results underscore the opposing functions of Notch signaling in the formation of PGCs and SSCs.

### Notch signaling functions similarly in the Bmp4 model and in vivo

**Fig. 4:**
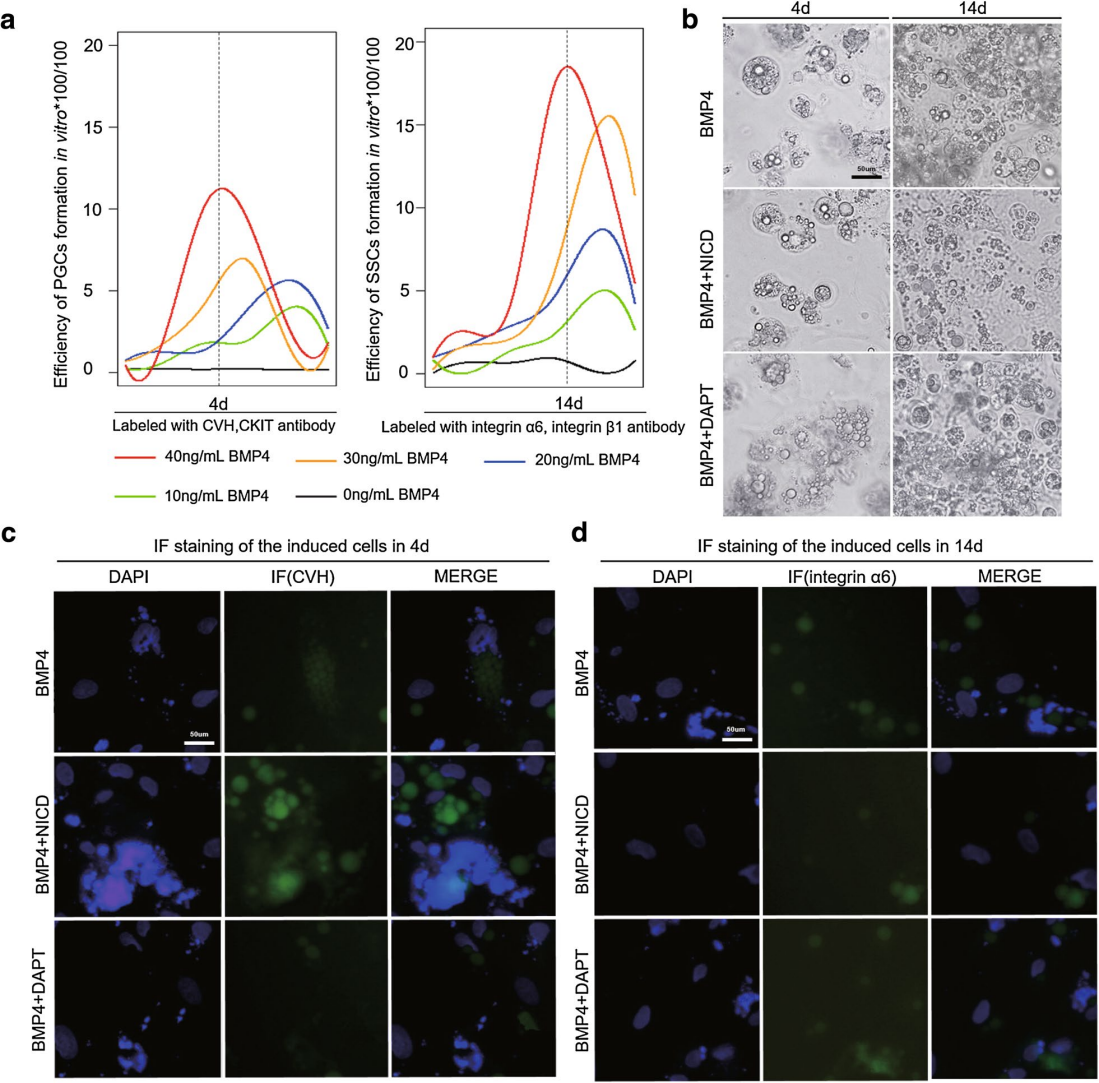
Notch signaling plays the same function in the BMP4 model as in vivo. **a** FACS was used to analyze the optimal time and efficiency of PGCs and SSCs induced by different BMP4 concentrations. PGCs appeared on the day 4, and SSCs appeared on day 14 when treated with 40 ng/mL BMP4. **b** The overexpression of NICD in the in vitro model promoted the formation of PGCs and inhibited the formation of SSCs. Inhibition of NICD yielded the opposite results in this model. Scale bar: 50 µm. **c**, **d** Indirect immunofluorescence was used to detect the formation efficiency of PGCs and SSCs after NICD overexpression and inhibition. Scale bar: 50 µm

Bone morphogenetic protein (BMP4) is an important endogenous factor for the origin and migration of germ cells [24]; however, this protein not only induces germ cell formation in vitro, but also regulates germ cell differentiation by interacting with Notch1 [25–27]. To study the function of Notch signaling in chicken germ cell differentiation in vitro, we established a model of BMP4-induced germ cell formation (Fig. 4a, Additional file 3: Figure S3). In our model, ESCs were treated with different concentrations of BMP4 (0–40 ng/mL), and none of these concentrations caused significant cytotoxicity to ESCs or affected cell proliferation status (Additional file 3: Figure S3).In general, the proliferation of the ESCs treated with the different BMP4 concentrations was similar. During the logarithmic growth phase (2–6 days), the cells continued to proliferate and reached the maximum peak on day 6. When the cells were in the plateau stage, the growth rate was stable for 8–10 days. The cell number stabilized on day 12 and began to decrease after 14 days due to apoptosis. Thus, BMP4 can induce germ cell formation without toxic effects that affect cell proliferation.

Different concentrations of BMP4, however, caused significant changes in cell morphology. A BMP4 concentration of 40 ng/mL resulted in embryoids on day 4, and the number of embryos increased gradually at 6–8 days. Then, the number of germ cells on day 14 was increased, and the number of reproductive cells was increased 12 days after the disintegration of the embryoid bodies (Additional file 4: Figure S4). The change of expression level of each marker also indicated that the concentration of 40 ng/mL was optimal for PGCs and SSCs generation in vitro as assessed by fluorescence activated cell sorting (FACS) (Fig. 4a).

**Fig. 5:**
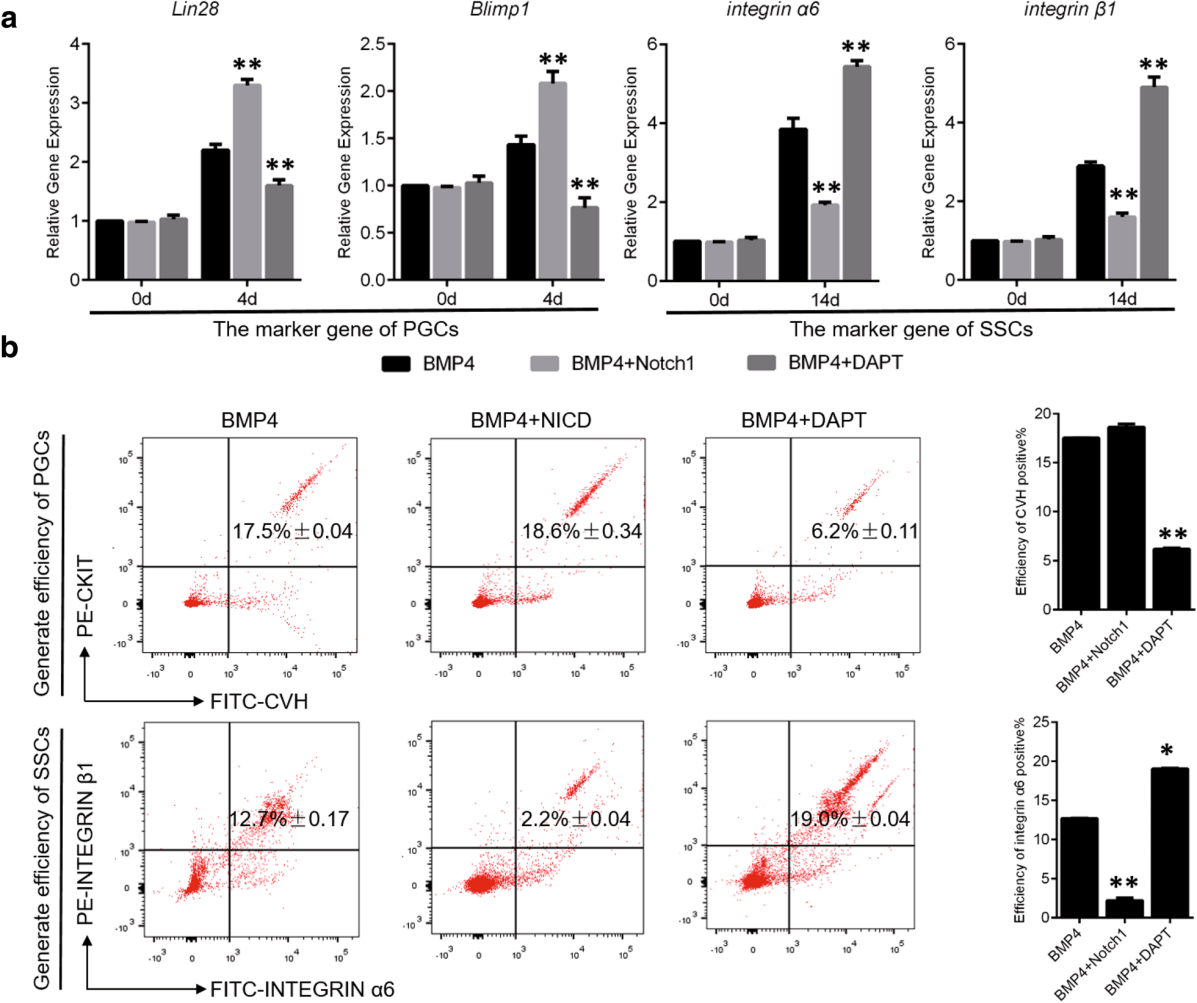
PGC and SSC formation efficiency analysis in the BMP4 model. **a** The expression levels of PGC and SSC marker genes were detected by qRT-PCR after NICD overexpression and inhibition on day 4 and 14. **b** PGC and SSC formation efficiency was analyzed by flow cytometry after NICD overexpression and inhibition in the in vitro model on day 4 and 14

Next, we examined inhibition and overexpression of NICD in the BMP4 induction model in vitro (Additional file 5: Figure S5A–C). Inhibition of NICD expression inhibited PGCs formation and promoted SSCs formation, while overexpression of NICD promoted the formation of PGCs and inhibited the formation of SSCs (Fig. 4b, c, d and Additional file 6: Figure S6). To validate the specific function of Notch signaling in vitro, we examined the expression of reproductive marker genes (Fig. 5a) and analyzed the effect of Notch signaling on germ cell formation before and after activation using FACS (Fig. 5b). The results of the reproductive marker gene expression and the efficiency of PGCs and SSCs generation were consistent with those observed in vitro (Fig. 5a, b). Together, these results demonstrate that Notch signaling plays opposing roles during the formation of PGCs and SSCs in both germ cells: Notch positively regulates PGC formation and negatively regulates SSC formation.

### High-throughput sequencing confirms a role for notch in the formation of PGCs and SSCs

**Fig. 6:**
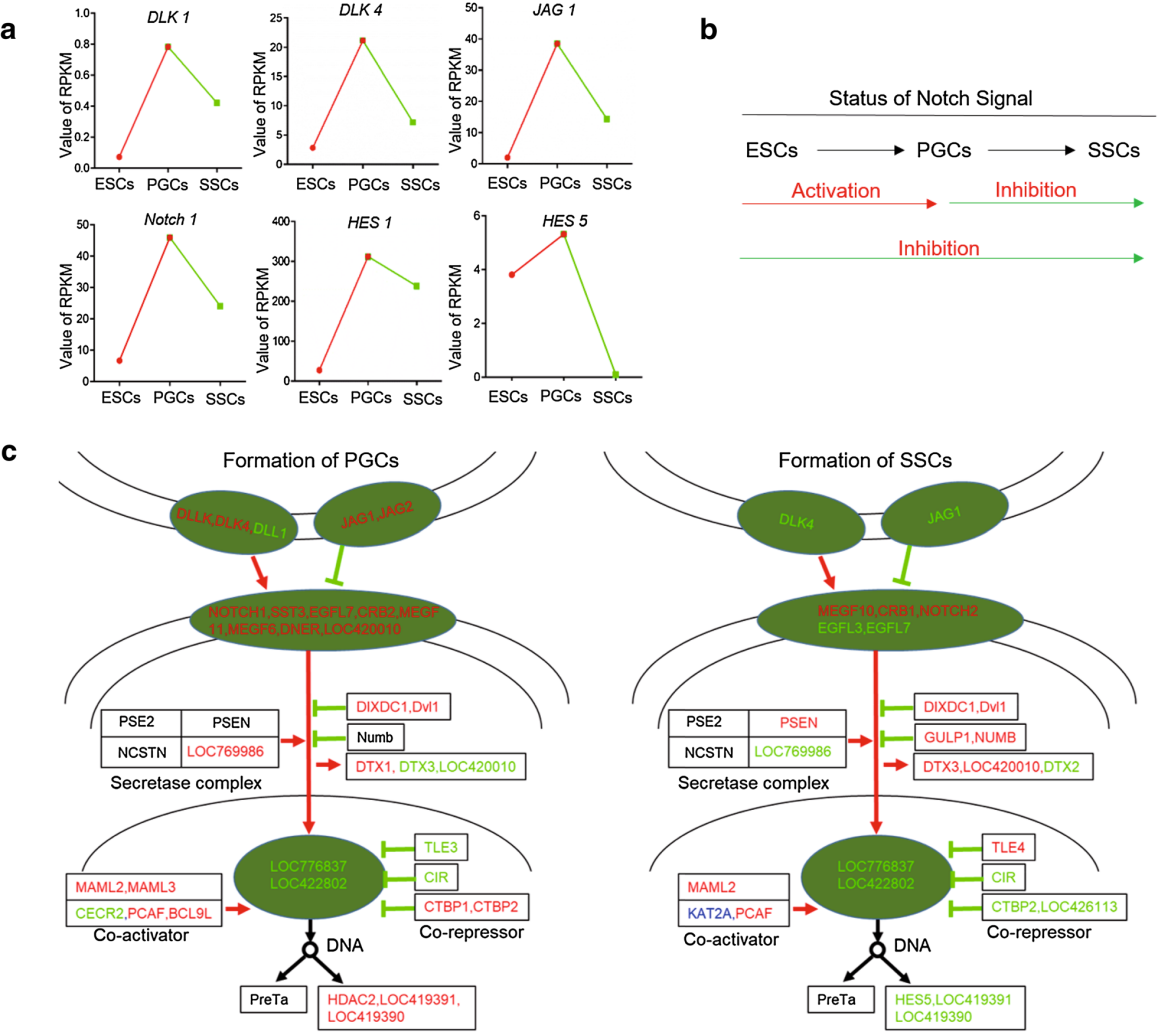
High-throughput sequencing results confirm the involvement of Notch signaling in the formation of PGCs and SSCs. **a** The key molecules of the Notch signaling pathway show opposite expression patterns during the formation of PGCs and SSCs. **b** Status changing of Notch signaling during PGCand SSC formation. **c** Transduction mechanism for the Notch signal in the formation of PGCs and SSCs

To analyze the specific functions of Notch signaling in PGCs and SSCs, we performed transcriptome sequencing of ESCs, PGCs, and SSCs (Additional file 7: Figure S7). The enrichment of differentially expressed genes in Notch signaling was dynamic (Fig. 6a). Specifically, Notch signaling was significantly upregulated during the process of ESC differentiation into PGCs. On the other hand, Notch signaling was downregulated during the process of PGC differentiation into SSCs. Interestingly, during ESC differentiation into SSCs, the Notch signal was enriched and expressed as a suppressive state (Fig. 6b). This indicates that the function of the signal is opposite during the process of PGCs and SSCs formation, which is consistent with the previous experimental results.

With respect to the two types of Notch ligands, Delta-like ligands and Serrate-like ligands, we found that the Delta-like ligands were expressed at their highest levels in PGCs. Conversely, the two Serrate-like ligand subtypes were expressed differently. Specifically, Jagged 1 was expressed at significantly higher levels in PGCs than in ESCs, and levels were decreased slightly in SSCs. The expression of Jagged 2 continued to decrease in all three kinds of cells. The expression of Notch receptors generally increased, with highest expression of Notch1 in PGCs. The expression of HES1 and HES5 was highest in PGCs and lowest in SSCs. Thus, these results illustrate the different functions of Notch signaling in the two germ cell formation processes.

In a previous study, we found that the dissociation of CBF-1/RBP co-suppression complexes was reversed albeit by unknown factors. Thus, we sought to elucidate the responsible factor using the high-throughput sequencing results. Based on the expression of the molecules in the Notch signaling pathway in PGCs and SSCs, we initially mapped the signal transduction mechanism of Notch signaling (Fig. 6c). We found that transducer like enhancer of split 3 (TLE3), TLE4, and C-terminal binding protein 2 (CTBP2) genes regulated the co-suppression CBF-1/RBP complex, and this reversal process is key to the reverse function of Notch signaling in the two types of reproductive stem cells. The expression of CBF-1/RBP complex in SSCs is reversed, which affects the differentiation of PGCs into SSCs.

## Discussion

In this study, we found that Notch signaling plays diametrically opposing roles in the formation of PGCs and SSCs. Moreover, upregulation of Notch1 expression during PGC formation facilitated the dissociation of the CBF-1/RBP complex and recruitment of MAML1 to form a coactivating complex, activating downstream transcription factor expression to promote PGC formation. In contrast, expression of TLE3, TLE4, and CTBP2 inhibited the dissociation of CBF-1/RBP complex and the expression of downstream transcription factors following downregulation of Notch1 in SSCs. These results were also confirmed using high-throughput sequencing.

Notch1 participates in Notch signaling to regulate the formation of PGCs and SSCs. The Notch signaling pathway is highly conserved through evolution and is involved in regulation of cell proliferation, differentiation, and apoptosis [28]. The Notch signal pathway relies mainly on four receptors: Notch1, Notch2, Notch3, and Notch4 [29]. In general, Notch1, Notch2, and Notch3 are expressed in tissues and organs of the central nervous system, mesoderm, hair, teeth, and kidney, while Notch4 is confined to mature macrophages, pancreas, and epithelial cells [30, 31]. Moreover, these receptors are also expressed during the development of germ cells. Specifically, Notch1, Notch2, and Notch3 are expressed in spermatogonia in mice, whereas only Notch2 molecules are expressed in the Sertoli cells. Furthermore, the Notch1 receptor was not detected in human testicular tissue [12]. In contrast to mammals, we found that Notch1 and Notch2 are expressed only during the germ cell development of chickens, and no significant differences were observed in Notch2 expression, indicating that Notch signaling during chicken germ cell formation is mainly regulated by Notch1. Furthermore, elucidation of the Notch signaling mechanisms that function in regulation of the fate of both germ cells in chicken revealed that the role of Notch in germ cell formation differs between species. SSCs develop from PGCs; however, we discovered that not only does Notch signaling promote the formation of PGCs, but this signaling pathway also plays an important, albeit opposing, role in the formation of SSCs.

Dissociation of the CBF-1/RBP transcription inhibition complex is a key factor in the formation of PGCs and SSCs. The classical Notch signaling pathway is a CBF-1/RBP-dependent pathway [32]. CBF-1/RBP is a transcriptional inhibitory factor that specifically binds to the DNA sequence “CGTGGGAA” and recruits SMRT, SKIP I/II, HDACs, and other proteins to form a co-inhibitory complex to prevent the transcription of downstream genes. Activation of the Notch signal resulted in CBF-1/RBP recruitment of specific factors to dissociate the co-suppressing complex and simultaneously recruited SKIP and MAML1 to form a co-activating complex to induce transcription of the downstream genes. The Notch signaling target genes are mostly members of the basic helix-loop-helix transcription factor family, including HES in mammals, XHey-1 in *Xenopus laevis*, and recently discovered BLBP [33, 34]. In this study, the co-inhibitory CBF-1/RBP complex dissociated during the formation of PGCs. The levels of HDAC1 and HDAC2, which inhibit the expression of genes [35, 36], were significantly reduced, and the co-activated complex was formed with MAML1. In contrast, co-activated complexes formed with MAML1 and CBF-1/RBP activated the expression of the downstream transcription factor HES1. Histone acetylation had no significant effect on the activity of the HES5 promoter, but significantly regulated the activity of the HES1 promoter. Although previous research showed that both HES1 and HES5 are involved in the regulation of PGCs and SSCs in mice [8] and our transcriptome sequencing confirms this notion, these results indicate that only HES1, and not HES5, is a downstream target of this process. We cannot exclude the possibility, however, that HES5 may be regulated by other epigenetic factors. At the same time, our results confirm that although there are differences in the levels of HDACs enrichment in the CBF-1/RBP complex during the formation of chicken PGCs and SSCs, However, both PGCs and SSCs can reduce the HDACs enzyme Activity after overexpression of NICD, Inhibition of NICD expression can increase HDACs enzyme activity. HDACs enzyme activity seems to have no direct relationship with the expression of HDACs themselves. Based on the results, we believe that the activity of HDACs in combination with their expression levels in the CBF-1/RBP complex participates in the regulation of downstream gene expression. As mentioned, we observed that the dissociation of the CBF-1/RBP co-suppression complex was reversed during differentiation of PGCs into SSCs. We speculated that this phenomenon was due to the alteration of expression of some unknown genes. We found that TLE3 [37, 38], TLE4 [39], and CTBP2 [40, 41] were the key genes that reversed the dissociation process of CBF-1/RBP co-suppression complex and led to the function of Notch signaling in the two reproductive stem cells.

The interaction between the Notch and BMP4 signaling pathways showed that a complex control network is involved in the process of differentiation from ESCs to SSCs and that different pathways participate in regulating this process via mutual interactions [42]. This study validated the role of multiple signaling pathways in the progress of chicken male germ cell differentiation in early stages, demonstrating that NOTCH and BMP4 signaling pathways work in coordination during differentiation. Specifically, this occurs through positive regulation of the BMP4 signaling pathway [43] and negative regulation of the NOTCH signaling pathway.

Using the gene chip approach, Zavadil et al. [44] identified components of the Notch pathway, including the basic transcription factor Hes1, which is a direct target of the Notch pathway. Recent research demonstrated a role for Hes1 in BMP4 regulation. The Delta pathway activates the proteolytic cleavage of NICD, followed by its translocation to the nucleus, increasing target gene expression via interactions with CSL (RBP-J/CBFI). SMAD3 increases the number of DNA-binding sites in CSL and NICD. NICD and SMAD3 interact directly, and SMAD3 is recruited to CSL-binding sites on DNA in the presence of CSL and NICD. These findings imply direct protein–protein interactions between the intracellular components of the NOTCH and BMP4 signaling pathways [45–47]. The Hes1 promoter in BMP4 stimulates transcription in cells overexpressing NICD, indicating Hes1 is a direct target of BMP4 signaling [42] and that BMP4-induced target cell antagonists may be used to prevent Hes1 activation [48].

## Conclusions

this work describes the opposing effects of the Notch signaling pathway on the generation of PGCs and SSCs in chicken and highlights the complexities in the signaling pathways involved in this regulation. Importantly, these findings will help researchers better interpret the mechanism of germ cell formation and at the same time provide new insights for clinical medicine in addressing human infertility issues.

## Methods

### Materials and reagents

Dulbecco’s modified Eagle medium (DMEM) and fetal bovine serum (FBS) were obtained from Gibco. Mitomycin-C was obtained from Roche. β-Mercaptoethanol, chicken serum, l-glutamine, sodium pyruvate, trypsin, collagen enzyme I, human stem cell factor (SCF), basic fibroblast growth factor (bFGF), human insulin-like growth factor (hIGF), and murine leukemia inhibitory factor (LIF) were acquired from Sigma-Aldrich. Antibodies specific to the following proteins were purchased: Oct4 (BioLegend, 1:100), Nanog (Abcam, 1:100), c-Kit (Southern Biotech, 1:100),CVH(Southern Biotech, 1:100), α6 and β1 integrins (Millipore, 1:100), and fluorescein isothiocyanate (FITC)-labeled goat anti-mouse IgM (Bio-Synthesis Inc., 1:100).

### Cell separation and culture

The isolation of ESCs was carried out from in vitro culture of blastodermal cells taken from the area pellucida of Stage X (EG&K) embryos. The isolated blastoderm cells were cultured in DMEM supplemented with 10% FBS, 1000 IU/ml LIF, 10 ng/ml bFGF, 10 ng/ml hIGF, and 5 ng/ml SCF at 37 °C in 5% CO_2_ with saturated humidity.

To obtain PGCs, chicken embryos were isolated from fertilized eggs on day 4.5 and rinsed with phosphate-buffered saline (PBS). The genital ridge of embryos was cut up and digested with 0.25% trypsin and 0.05% EDTA for 5–10 min. Cells were filtered and differentially cultured in dishes for 30 min in TCM-199 medium containing 10% FBS.

The testis was obtained from fertilized eggs at day 18.5 and dissected to obtain chicken SSCs. The tissue was digested for 30 min with collagenase and then with trypsin. The cells were cultured in DMEM supplemented with 10% FBSat 37 °C in 5% CO_2_ with saturated humidity and passaged every 2–3 days.

### Overexpression and inhibition of active Notch1 receptor (NICD)

The coding sequence of the Notch1 receptor was obtained according to the accession number NM_001030295.1, and the NICD sequence was determined to be 5214–7686 bp. The NICD sequence was inserted into the pcDNA3.0 vector(pcDNA3.0-NICD) by chemical synthesis. The specific sequencing primer was as follows: GCGTGCTGTCCCCA GTGGACTC. DAPT is a specific inhibitor of Notch1 receptor and was used to effectively inhibit the process of Notch1 receptor cleavage into NICD.

### In vivo and in vitro experimental design

The Notch1 inhibitor (DAPT) and overexpression vector were injected into eggs by tip injection with paraffin sealing and incubated at 38.5 °C. The experimental groups were sampled at 5.5 and 18 days, respectively, as detailed in Additional file 8: Table S1. To assess the second-generation chicken ESCs, the experimental groups were subjected to induction experiments as described in Additional file 9: Table S2. The cells were replaced with new culture medium every 2 days, and the morphological changes of the cells were observed by inverted microscope. During the induction process, the cells were sampled every 2 days.

### Western blot analysis

Genital ridges and testes were collected 5.5 and 18 days after the eggs hatched. Cells were sampled 4 and 14 days after induction. RIPA buffer was used to lyse cells and to extract the proteins. Total cellular protein (20 μg) was mixed with 5 μL sample buffer and boiled for 3–5 min to denature the proteins. The proteins were separated by 10% SDS-PAGE and transferred to nitrocellulose membranes in a semidry manner, followed by blocking with Tris-buffered saline containing Tween and 5% FBS for 1 h at room temperature. The membranes were incubated with the primary antibodies against NOTCH1 overnight at 4 °C. After the membranes were washed, the corresponding secondary antibodies were added. The membranes were then incubated at 37 °C for 2 h. Bands were visualized using a DAB substrate kit to detect horseradish peroxidase.

### Co-immunoprecipitation (Co-IP)

Following collection of the PGCs and SSCs and the extraction of protein, the protein was incubated with RBP antibody overnight. The samples were then centrifuged at 1000×*g* at 4 °C for 10 s. The protein was collected and subjected to western blot experiments using HDAC1, HDAC2, and MAML1antibodies as described above.

### Periodic acid-schiff (PAS) staining

The reproductive ridges of each in vitro group were fixed overnight with Rossman’s fixative solution. The samples were then treated by dewatering, dipping, embedding, waxing, slicing, putting on slides, dewaxing, and PAS staining. After these steps, the morphological changes of gonadal gland and the number of PGCs were observed using an Olympus microscope.

### Quantitative reverse transcription-PCR (qRT-PCR)

Total RNA was extracted from tissues and cells. cDNA was synthesized by reverse transcription. Fluorescent quantitative PCR was used to detect the gene expression of NOTCH1, Lin28, Blimp1, integrin α6, and integrin β1. The reaction volume for PCR amplification included 2 μL cDNA, 10 μL SYBRTaq, 0.8 μL each of upstream and downstream primers, 0.4 μL RoxII, and double distilled water to make up the volume of 20 μL (Primes in Additional file 10: Table S3).

### Indirect immunofluorescence

After induction, cells were fixed with 4% paraformaldehyde for 30 min, rinsed with PBS three times, and then permeabilized with 0.5% Triton X-100 for 10 min. The sections were then washed with PBS again three times and blocked with 10% bovine serum albumin (BSA) in PBS for 30 min at room temperature. Then, the primary antibodies against CVH and integrin α6 were added, and the sections were incubated overnight at 4 °C. After 3 rinses with PBS-Tween (PBST), the corresponding secondary antibody was applied, followed by incubation in the dark at 37 °C for 1 h. After 3 washes with PBST, DAPI was used for nuclear staining. The sections were observed by fluorescence microscopy.

### FACS

The genital ridge and testis samples were digested with trypsin to yield cell suspensions. In addition, induced cells were collected, and 1 mL of the cell suspension (1 × 10^6^ cells) was aliquoted into 1.5-mL centrifuge tubes. Centrifugation was carried out at 1500 rpm for 5 min, after which the supernatant was discarded. The cells were washed with PBS, and 200 μL (1:200 dilution) of fluorescein-labeled antibody (c-Kit and CVH antibody for labeling PGCs, integrin α6 and integrin β1 antibody for labeling SSCs) was added, followed by incubation at 4 °C for 1–2 h. Next, the suspension was centrifuged, and the supernatant discarded. Precooled PBS was added, and cells were washed twice to remove excess unbound antibody. Then, 500 μL precooled PBS was added, and samples were mixed by blowing and tapping. Antibody binding was then detected using FACS.

### Statistical analysis

All data are provided as mean ± SEM, and the differences between groups were analyzed by Student’s *t* tests and one-way ANOVAs. A *p*-value of < 0.05 was considered statistically significant.

## Additional files

**Additional file 1: Figure S1.** Effects of key signal molecules in Notch signaling duringgonadal development. A. Diagram of construction of NICD overexpression vector. B. Western blot results show that transfection of DF-1 chicken fibroblasts with pcDNA3.0-NICD significantly increases the level of NICD expression. C. Western blot results show that DAPT does not affect Notch2 expression. D. Reproductive ridge development. Nor, normal; UN, not normal.

**Additional file 2: Figure S2.** Histone acetylation regulates HES1 gene expression. A. CHIP-qPCR shows that the RBP complex has stronger binding ability to the HES1 promoter and weaker binding ability to the HES5promoter. B. Dual fluorescein reporter enzyme shows that TSA inhibited HDAC-upregulated HES1 promoter activity but not HES5 promoter activity. ## *p *< 0.01 compared to PGL3-basic. &&*p *< 0.01compared to PGL3-HES1 group. ** *p *< 0.01 compared to the PGL3-HES1 group and the PGL3-HES1 + TSA group.

**Additional file 3: Figure S3.** Assessment of different concentrations of BMP4(0-40 ng/mL) on cytotoxicity and cell proliferation of ESCs.

**Additional file 4: Figure S4.** Morphological changes during induction of ESCs to germ cells with 0-40 ng/mL BMP4. The concentration of BMP4 at 40 ng/mL had the greatest effect on the morphological changes of ESCs. Scale bar: 50 µm.

**Additional file 5: Figure S5.** Overexpression or inhibition of NICD affects the expression of Notch1 in the BMP4-induced model. A. Schematic diagram of in vitro induction experiments. B, C. DAPT inhibits the expression of NICD in the BMP4-induced model, and overexpression of NICD restores the expression level of Notch1.

**Additional file 6: Figure S6.** Changes in the number of embryoid bodies in the *in vitro*BMP4-induced model after overexpression and inhibition of NICD expression.

**Additional file 7: Figure S7.**  Sequencing results of ESCs, PGCs, and SSCs transcriptome.

**Additional file 8: Table S1.** Experiment grouping in vivo.

**Additional file 9: Table S2.** Induction grouping in vitro.

**Additional file 10: Table S3.** The sequences of qRT-PCR primers.

## Abbreviations

CoIP: co-immunoprecipitation
ESCs: embryonic stem cells
FACS: fluorescence activated cell sorting
PCAF: the p300/CBP-related factor
PGCs: primordial germ cells
SSCs: spermatogonial stem cells
