# Supplementary material for: NICD-mediated notch transduction regulates the different fate of chicken primordial germ cells and spermatogonial stem cells
Source: Cell Biosci. 2018 Jun 19;8:40. doi: 10.1186/s13578-018-0238-y (PMC6009047; doi:10.1186/s13578-018-0238-y)
Supplement: Supplementary file 8 — Additional file 8: Table S1. Experiment grouping in vivo. [file 13578_2018_238_MOESM8_ESM.docx]

Table S1 Experiment grouping *in vivo*

| Group | ddH_2_O | DMSO | OE-Notch1（12ug/100ul） | DAPT（10nM/L） |
| --- | --- | --- | --- | --- |
| BLANK | - | - | - | - |
| CON | - | + | - | - |
| OE-Notch1 | - | - | + | - |
| DAPT | - | - | - | + |

Note: DAPT is an inhibitor of Notch signaling pathway. Effective concentration of DAPT was 10nM/L. OE-Notch1 was a Notch1 overexpressing vector. Vector and inhibitors of the injection dose was 100ul. DMSO was used to dissolve DAPT.
