# Supplementary material for: NICD-mediated notch transduction regulates the different fate of chicken primordial germ cells and spermatogonial stem cells
Source: Cell Biosci. 2018 Jun 19;8:40. doi: 10.1186/s13578-018-0238-y (PMC6009047; doi:10.1186/s13578-018-0238-y)
Supplement: Supplementary file 9 — Additional file 9: Table S2. Induction grouping in vitro. [file 13578_2018_238_MOESM9_ESM.docx]

Table S2 Induction grouping *in vitro*

| Group | DMEM | BMP  （40 ng/mL） | OE-Notch1（1ug/500ul） | DAPT（10nM/L） |
| --- | --- | --- | --- | --- |
| CON | + | - | - | - |
| BMP4 | + | + | - | - |
| DAPT | + | + | + | - |
| OE-Notch1 | + | + | - | + |
