# Supplementary material for: NICD-mediated notch transduction regulates the different fate of chicken primordial germ cells and spermatogonial stem cells
Source: Cell Biosci. 2018 Jun 19;8:40. doi: 10.1186/s13578-018-0238-y (PMC6009047; doi:10.1186/s13578-018-0238-y)
Supplement: Supplementary file 10 — Additional file 10: Table S3. The sequences of qRT-PCR primers. [file 13578_2018_238_MOESM10_ESM.docx]

Table S3 The sequences of qRT-PCR primers

| Gene | Primer Sequence（5'-3'） | | Genebank |
| --- | --- | --- | --- |
|  | F | R |  |
| *Notch1* | TCTTGCTTGCCTTCATTGGA | GCGCTTCTTCTTGCTCGACT | NM_001030295.1 |
| *Notch2* | TCTGGCTGGACTGGTGCTTA | ATTGAGGCAATGACCGGAGT | NM_001252033.1 |
| *integrinβ1* | TGTTTGTGGGGACCAGATTG | CCAGGTGACATTTCCCATCA | XM_015289566.1 |
| *I**ntegrinα6* | GCTGGAAACATGGACCTGGATAA | TTCAGGTCAAGTTTGTCAGGCTGTA | NM_205289.1 |
| *Lin28* | CTGGCATCTGTAAGTGGTT | GAAGCCCTCCATGTGC | NM_001031774.2 |
| *Blimp-1* | CCAAGAATGCGAACAGA | TGGAGGAGGGATAGTGC | AB434917.1 |
| *MAML1* | AAGAAGCTGCGTCGTGATGA | GCTGCAAGGTATCTCCACTGC | XM_414607.2 |
| *MAML2* | CAGCAGATGTTGGCAGAAGC | TGCTTGTTGCATGTTGACCA | XR_027136.1 |
| *MAML3* | TGCTGAGCTGAACCAGAGGA | GCCATCTGAGCTTGCATCTG | XM_420411.2 |
| *PCAF* | CATGATGTGGCTGGTTGGTT | TCGGAAGCAGATACCACCAAT | XM_426001.2 |
| *HES1* | GGTGAAGCACCTGAGGAACC | GAACCTCGTCACCTCGTTCA | NM_001005848.1 |
| *β-actin* | CAGCCATCTTTCTTGGGTAT | CTGTGATCTCCTTCTGCATCC | L08165.1 |
